# Magnetically driven hydrogel microrobots for enhancing the therapeutic effect of anlotinib on osteosarcoma

**DOI:** 10.3389/fbioe.2024.1409988

**Published:** 2024-11-13

**Authors:** Haoyu Wang, Haitian Jiang, Yining Tao, Binghui Yang, Jiakang Shen, Haoran Mu, Chongren Wang, Xiyu Yang, Zhengdong Cai, Mu Li, Wei Sun, Liu Yang, Mengxiong Sun

**Affiliations:** ^1^ Department of Orthopedic Oncology, Shanghai General Hospital, School of Medicine, Shanghai Jiao Tong University, Shanghai, China; ^2^ Shanghai Bone Tumor Institution, Shanghai, China; ^3^ State Key Laboratory of Robotics and System, Harbin Institute of Technology, Harbin, China; ^4^ Department of Pharmacy, Second Affiliated Hospital of Harbin Medical University, Harbin Medical University, Harbin, China

**Keywords:** microrobots, magnetic-driven, drug delivery system, VEGFR, met, osteosarcoma

## Abstract

**Introduction:**

Osteosarcoma, characterized by high mortality and disability rates, poses a significant challenge due to its complex genetic background and the absence of specific membrane receptors, which hinder effective targeted therapy. Active targeting has emerged as a promising approach to address this issue.

**Methods:**

In this study, magnetically driven hydrogel robots (MMHR) were utilized to load and deliver drugs precisely to target sites. The drugs included SCR1481B16, a specific MET inhibitor proven to inhibit MET-driven tumor growth, and Anlotinib. The microrobots were designed to navigate under magnetic guidance, enhancing drug efficacy while minimizing damage to normal tissues.

**Results:**

The study explored the potential of MMHR loaded with SCR1481B16 and Anlotinib in the treatment of Anlotinib-resistant osteosarcoma. The microrobots were successfully designed and produced, demonstrating the ability to deliver drugs precisely to tumor sites. Evaluation of the microrobots showed an enhanced sensitivity of tumors to Anlotinib, providing new insights into the treatment of drug-resistant osteosarcoma.

**Discussion:**

Tumors overexpressing MET often develop resistance to VEGFR-targeted drugs. The use of SCR1481B16 as a MET inhibitor in combination with Anlotinib, delivered by magnetically driven hydrogel microrobots, offers a novel strategy to overcome this resistance. However, further in-depth research and validation are required before the clinical application of this method can be considered.

**Conclusion:**

In conclusion, magnetically driven hydrogel microrobots loaded with SCR1481B16 provide a promising new strategy for enhancing the sensitivity of Anlotinib-resistant osteosarcoma, bringing hope for future clinical applications in the treatment of this challenging disease.

## 1 Introduction

Osteosarcoma is the most common highly malignant bone tumor (Kansara et al., 2014). The primary treatment method is limb-salvage surgery combined with neoadjuvant chemotherapy. However, up to 90% of patients still develop drug resistance (Gill et al., 2013; Pawson et al., 2014). Patients who fail first-line treatment can only rely on second-line chemotherapy or targeted drugs to prolong their lives. However,second-line drugs also lead to treatment failure due to drug resistance, urgently requiring new therapeutic agents and combination treatment strategies.

Targeting VEGFR to inhibit tumor angiogenesis has always been a research hotspot in the field of tumor therapy. The VEGFR family includes VEGFR1 (FLT-1), VEGFR2 (KDR), and VEGFR3 (FLT-4). VEGFR1/2 are mainly expressed in vascular endothelial cells, with VEGFR2 being the primary receptor for VEGF. Activation of VEGFR2 triggers downstream signal transduction, promoting tumor angiogenesis (Apte et al., 2019). Anlotinib, a multi-target TKI (tyrosine kinase inhibitor) small-molecule targeted drug with VEGFR as its primary target, has been clinically approved for the treatment of soft tissue sarcomas (Chi et al., 2018). Our team’s previous research has shown satisfactory results of Anlotinib in preclinical and clinical studies of osteosarcoma (Wang et al., 2019). The tolerability of Anlotinib is similar to other tyrosine kinase inhibitors targeting VEGFR and other tyrosine kinase-mediated pathways. Compared to Sunitinib, the incidence of grade 3 or higher side effects is significantly lower with Anlotinib. Unfortunately, we have observed in clinical treatment that some osteosarcoma patients gradually develop resistance to Anlotinib, leading to disease progression. Current research on Anlotinib resistance mainly focuses on genes promoting survival mutations in tumor cells. For example, in lung cancer, upregulation of BMP4 and HSPG2 by the transcription factor enhancer-binding protein TFAP2A reduces the sensitivity of tumor cells to Anlotinib (Zhang et al., 2020). In non-small cell lung cancer, transcriptome analysis of Anlotinib-resistant cell line NCI-H1975 revealed a strong correlation between CXCL2 and Anlotinib resistance (Lu et al., 2019). Researchers have also noted the important role of MET in acquired resistance to tumor TKI targeting. In lung cancer, Jeffrey A. Engelman et al.found that MET can lead to gefitinib resistance in lung cancer by activating ERBB3 signaling (Engelman et al., 2007). Similarly, in liver cancer, Kexun Zhou et al. found that combining MET inhibitors with treatment for advanced hepatobiliary cancer patients can effectively improve their acquired resistance, significantly enhancing their response to avolitinib (Zhou et al., 2023). This suggests that MET can serve as a therapeutic target for TKI treatment while high MET expression may also be a cause of tumor TKI targeting resistance. Combining MET inhibitors may be a potential approach to improving the efficacy of Anlotinib.

Although Anlotini’s side effects are less severe than those of Sunitinib, clinical trials have shown that the incidence of common adverse events associated with Anlotinib still exceeds 30%, including hypertension (34%), proteinuria (67%), hand-foot skin reaction (53%), hypothyroidism (57%), and others (Li, 2021). Cerebral infarction, hemoptysis, and severe liver or kidney dysfunction are contraindications for Anlotinib use. Furthermore, animal experiments have also demonstrated the reproductive toxicity and teratogenicity of Anlotinib, excluding pregnant women from the treatment population (Chinese Association for Clinical et al., 2020). Therefore, there is a need for reasonable approaches to improve the safety of Anlotinib, reduce contraindications, and expand the beneficiary population.Efficient drug delivery systems are considered one of the effective means to enhance the effectiveness and safety of tumor treatment. Drug delivery systems (DDS) are technological systems that can control the distribution of drugs in the body in a spatiotemporal and dosage-specific manner. DDS can improve the targeting and selectivity of drugs, enhance their stability and bioavailability, and achieve synergistic and multi-dimensional treatment, thereby improving the effectiveness and safety of tumor treatment (Vargason et al., 2021; Yi et al., 2022; Li et al., 2024).As an innovative drug delivery technology, micro-nano robotic drug delivery systems can accurately deliver drugs to specific diseased sites by driving nanoscale robots with external energy or self-navigation mechanisms (Soto et al., 2020). Currently, diverse driving modes have been developed for microrobots, including ultrasound (Xu et al., 2014; Ahmed et al., 2016), photothermal (Dai et al., 2016; Wu et al., 2014), self-propelled (Ma et al., 2015; Wan et al., 2019), and electric fields (Calvo-Marzal et al., 2010; Yin et al., 2024a; Yin et al., 2024b), enabling them to achieve autonomous movement and precise navigation in complex biological environments such as blood vessels, lymphatics, and tissues, thus greatly enhancing the targeting and selectivity of drugs (Choi et al., 2021).These micro-nano robots possess the capability to carry multiple drugs or biological agents simultaneously and flexibly adjust the release sequence and proportion of drugs, thereby exerting synergistic effects among drugs and effectively overcoming drug resistance and side effects. Among the various microrobots, magnetic-driven hydrogel microrobots (MMHR) stand out due to their unique advantages. These hydrogel-based capsule-shaped microrobots can achieve precise motion control and navigation with the aid of an external magnetic field, effectively overcoming the fluid dynamic resistance and interference caused by Brownian motion in low Reynolds number fluids (Chen et al., 2021; Yin et al., 2023). Moreover, the high water absorption and porosity of hydrogels enable magnetic-driven hydrogel microrobots to efficiently carry drugs and achieve responsive drug release, effectively preventing premature drug leakage and nonspecific effects. The high biocompatibility and biodegradability of hydrogels ensure high safety and bioavailability for this drug delivery system while reducing tissue cell toxicity and side effects (Zhang et al., 2021; Hu et al., 2020; Yin et al., 2022). In summary, micro-nano robotic drug delivery systems, especially magnetic-driven hydrogel microrobots, exhibit tremendous application potential and value in the field of drug delivery due to the harmlessness and high controllability of the added external magnetic field to the human body. Given the promising prospects of magnetic-driven hydrogel microrobots in drug delivery systems, it is of great significance to explore their application value in the diagnosis and treatment of osteosarcoma.

Micro-nano robots capable of control and regulation are considered to have innovative potential in precision targeted drug delivery systems. To explore the possibility of magnetic-driven hydrogel microrobots loaded with MET inhibitors in enhancing the efficacy of TKI targeted drugs against osteosarcoma, we have chosen SCR1481B1, a MET inhibitor, as the research drug. By loading it together with Anlotinib onto magnetic-driven hydrogel microrobots, we have enhanced the killing effect on osteosarcoma cells *in vitro*.Our research aims to investigate the application value and progress of magnetic-driven hydrogel microrobots loaded with SCR1481B1 in improving the sensitivity of osteosarcoma to Anlotinib, aiming to provide new ideas for expanding the population benefiting from antitumor drugs and improving the prognosis of osteosarcoma patients.

## 2 Materials and methods

### 2.1 Cell lines, reagents and cell culture

The human osteosarcoma cell lines U2OS, DUNN, SaOS-2, and HOS were cultured in an incubator with 5% CO2 and 95% air at 37°C, supplemented with 10% fetal bovine serum (FBS; Wisent, Canada) and 1% penicillin-streptomycin-glutamine (Wisent Inc., Canada). Cells were passaged using TrypLETM Express (Gibco, Thermo Fisher Scientific Inc., United States) when they reached 90%–100% confluence. Cell line authentication for *in vitro* and *in vivo* studies was performed using short tandem repeat (STR) DNA profiling, and all cell lines were stored at the Shanghai Institute of Bone Tumor Research (Shanghai, China). The reagents used in the study included SCR1481B1 (# HY-18711A, United States) and Anlotinib (Selleck #S8726, CN). Antibodies used in this study were purchased as follows: anti-VEGFR2 (MCE # HY-P81643,1:1000), anti-β-actin (MCE #HY-P7453,1:1000). HRP-conjugated goat anti-rabbit IgG (MCE#L3042, 1:5000) and goat anti-mouse IgG (MCE#101, 1:5000) were used for protein blotting.

### 2.2 Colony formation

The passaged osteosarcoma cells were washed twice with PBS and plated onto 60 mm cell culture dishes at a density of 3,000 cells per dish. The osteosarcoma cells were then treated with or without drugs for 1 week. Finally, the cell colonies were stained using crystal violet staining solution (Beyotime, China) according to the manufacturer’s instructions.

### 2.3 Calceim/AM-P

To assess cell viability, osteosarcoma cells were plated onto 24-well plates at a density of 5000 cells per well. The osteosarcoma cells were then treated with or without drugs for 3 days. Osteosarcoma cells were stained with calcein-AM and propidium iodide (PI) (Beyotime, China) according to the manufacturer’s instructions. Briefly, cells were washed with PBS and then incubated with 2 μM calcein-AM and 4.5 μM PI in PBS at 37°C for 30 min. After incubation, cells were washed with PBS and immediately observed under a fluorescence microscope. Live cells were identified by green fluorescence (calcein-AM), while dead cells were identified by red fluorescence (PI). Images were captured using a Leica TCS SP eight confocal laser scanning microscope (Leica, United States) and processed with Leica Application Suite X (LAS X; Leica, United States).

### 2.4 CellTiter-Glo luminescent cell viability assay

Using the CellTiter-Glo Luminescent Cell Viability Assay (Promega, United States), 3D-cell viability was evaluated according to the manufacturer’s instructions. Briefly, cells were plated at a density of 2 × 104 cells per well in a 96-well plate and incubated for 24 h. After treatment with or without drugs for 5 days, 100 μL of CellTiter-Glo reagent was added to each well and mixed for 2 min on an orbital shaker to induce cell lysis. The plate was then incubated at room temperature for 10 min to stabilize the luminescent signal. Luminescence was detected using a SpectraMax M3 microplate reader (Molecular Devices, United States).

### 2.5 Three-dimensional cell culture

The cell precipitate was resuspended in a three-dimensional cell culture medium composed of the following components: DMEM (Thermo Fisher Scientific, United States), supplemented with 1% penicillin-streptomycin (Gibco, United States), 10% fetal bovine serum (FBS; Wisent, Canada), 0.5% GlutaMax (Gibco, United States), 0.5% N2 supplement (Thermo Fisher Scientific, United States), 0.5% MEM non-essential amino acid solution (Thermo Fisher Scientific, United States), 0.5% B-27^®^ serum-free supplement (Thermo Fisher Scientific, United States), 8 mg/mL EGF (PeproTech, United States), 10 ng/mL bFGF (MCE, MedChemExpress, United States), 10 ng/mL IGF-1 (MCE, MedChemExpress, United States), and 5 ng/mL TGF-β 3 (MCE, MedChemExpress, United States). The prepared cell suspension was then added to a U-bottom 96-well plate with ultra-low attachment (Corning, United States). The cell count in each well was adjusted to 300 to 5000 cells. The plate was then placed in a 37°C incubator with a 5% carbon dioxide concentration. Each well received 200 μL of culture medium, which was replaced every 24–36 h.

### 2.6 Western blotting

Proteins were extracted for total protein using Radioimmunoprecipitation Assay (RIPA) lysis buffer (Beyotime, China). Tissue was homogenized in RIPA lysis buffer (Beyotime, China) containing protease and phosphatase inhibitors. Cells were washed with PBS and lysed in RIPA buffer. The cell lysate was centrifuged at 12,000 g for 30 min at 4°C, and the supernatant was collected. Protein concentration was measured using the Pierce BCA Protein Assay Kit (#23325, Thermo Fisher Scientific, United States) and a SpectraMax M3 microplate reader (Molecular Devices, United States). Proteins were separated by Sodium Dodecyl Sulfate Polyacrylamide Gel Electrophoresis (SDS-PAGE) and transferred to a 0.45 μm polyvinylidene difluoride (PVDF) membrane using a Mini-PROTEAN Tetra Vertical Electrophoresis Cell with PowerPac HV Power Supply (Bio-Rad, United States) and Mini trans-Blot Module for tank transfer systems (Millipore, United States). The membrane was blocked with 5% non-fat milk in Tris-buffered saline solution containing 0.1% Tween-20 (TBS-T) for 1 h at room temperature, followed by incubation with specific primary antibodies overnight at 4°C. Subsequently, the membrane was washed three times with TBS-T for 10 min each and then incubated with an HRP-conjugated secondary antibody for 1 h at room temperature. Actin was used as a protein loading control. Protein signals were detected using SuperSignal West Femto Maximum Sensitivity Substrate (Thermo Fisher Scientific, United States) and imaged using a chemiluminescence imaging system, Amersham Imager 600 (GE Healthcare, United States) and Tanon 5200 (Tanon, China).

### 2.7 Synthesis of a magnetically driven hydrogel micro-robot drug delivery system

This study utilized microfluidic technology to fabricate hydrogel microrobots using microdroplets. In droplet microfluidics, the formation of droplets primarily depends on the tension difference between the continuous phase and the dispersed phase fluids. Under the combined action of surface tension and shear force, droplets are formed at the interface of the two phases. A schematic diagram of the preparation of gelatin microspheres using a flow focusing device is shown in Figure 2B. Specifically, vegetable oil was taken and Tween-20 was added to obtain a continuous phase fluid (1000:1). 333 mg of gelatin (gel strength: ∼250 g Bloom, Aladdin, China) was dissolved in 7 mL of pure water, and 20 mg of the photoinitiator 2-hydroxy-4′-(2-hydroxyethoxy)-2-methylpropiophenone was dissolved in 3 mL of ddH2O. The solutions were uniformly mixed to obtain 10 mL of a gelatin aqueous solution with photoinitiator. 0.9 mL of the gelatin aqueous solution with photoinitiator, 0.15 mL of DOX solution, and 0.15 mL of RO-3306 solution were mixed uniformly. Then, 0.15 mL of a 25% mass fraction nano-iron oxide dispersion was added. The mixture was placed on a test tube oscillator for uniform mixing to obtain the dispersed phase fluid required for the experiment. Two 10 mL disposable syringes were taken, and 10 mL of the continuous phase fluid was aspirated into each syringe. Then, 1 mL of the dispersed phase fluid was aspirated. The flow rates of the microinjection pumps controlling the continuous phase fluid and the dispersed phase fluid were adjusted, and both microinjection pumps were started simultaneously. The dispersed phase fluid and the continuous phase fluid were injected from the syringes through plastic capillaries into the flow focusing device. When uniform microdroplets were formed at the outlet of the device, the plastic capillary at the outlet of the microchannel was placed in the oil phase of the collection beaker to obtain unsolidified magnetic drug-loaded gelatin microspheres. After collection, the beaker was placed under a UV lamp for 20 min to solidify the hydrogel. The solidified gelatin microspheres were placed in a test tube, centrifuged, and washed three times to remove the oil phase, obtaining a dispersion of magnetic gelatin microrobots. The dispersion was covered with tin foil to block light and stored in a refrigerator at 4°C.

### 2.8 Setup of external magnetic field

The magnetic field generator consists of a three-degree-of-freedom Helmholtz coil, a multi-function data acquisition unit (DAQ, NI-PCI-6259), and three single-channel output power amplifiers. By controlling the current and voltage of the Helmholtz coil through a drive signal amplified by a voltage amplifier, an external rotating uniform magnetic field can be generated in three-dimensional space to manipulate the hydrogel microrobots. The Helmholtz coil is located on the observation platform of the microscope, enabling real-time observation of the swimming hydrogel microrobots.

A series of controlled experiments were conducted to investigate the locomotor performance of the microrobots under the influence of an external magnetic field, aiming to clarify the effects of different driving frequencies and magnetic field strengths on the speed of the microrobots. Subsequently, a control strategy utilizing a three-dimensional rotating magnetic field generated by the three-degree-of-freedom Helmholtz coil was employed to navigate the microrobots. In the x-z plane, the microrobots moved around the short axis (*x*-axis) under the application of a magnetic field given by H(t) = H0 [cos (ωt)ex + sin (ωt)]ez (where H0 is the magnitude of the magnetic field, ω is the angular frequency, and t is time). Here, ex and ez represent unit vectors along the *x* and *z*-axes, respectively (likewise, ey represents the unit vector along the *y*-axis in the following context). In the y-z plane, microrobots moved around the long axis (*y*-axis) under the application of a magnetic field H(t) = H0 [cos (ωt)ey + sin (ωt)]ez. By controlling the input of current, the direction of the rotating magnetic field can be altered to steer the microrobots. Based on this dynamic control scheme, magnetically controlled microrobots can follow predefined trajectories.

### 2.9 *In vitro* simulation of drug loading inhibition of magnetically driven hydrogel micro robot

We placed the microrobots loaded with SCR1481B1 and Anlotinib into a cell culture dish (35 mm in diameter) containing complete culture medium. Subsequently, a directional magnetic field was applied to the robots for 2 min to guide them to the center of the dish. This process simulated the navigation capability of the microrobots and their precision drug delivery system. For cells cultured under two-dimensional conditions, we used PI staining to detect cellular responses 72 h after drug release. Specifically, after aspirating the culture medium and washing the cells with PBS, we incubated them in a PBS solution containing 4.5 μM PI at 37°C for 30 min. After incubation, the cells were washed with PBS and immediately observed under a fluorescence microscope. Dead cells were identified by red fluorescence (PI). Imaging was performed using a Leica TCS SP8 laser scanning confocal microscope (Leica, United States), and captured images were processed using Leica Application Suite X (LAS X; Leica, United States). For cells grown under three-dimensional conditions, we captured green fluorescent images using the Leica TCS SP8 laser scanning confocal microscope and processed them using the Leica Application Suite X (LAS X). Additionally, we employed the CellTiter-Glo luminescent cell viability assay (as previously described) to assess cell viability under three-dimensional culture conditions.

### 2.10 Statistical analysis

The statistical analysis details of various experiments are described in the relevant methods. If not specified, GraphPad Prism eight software (GraphPad software, United States) is used for statistical analysis. After confirming that the values follow a normal distribution, two-tailed Student’s t-test is used to determine the significance of the difference between two independent samples. Pearson correlation analysis and Spearman correlation analysis are used to determine the correlation between two sets of variables.

## 3 Result

### 3.1 The response of osteosarcoma cells to anlotinib is related to the level of MET expression

**FIGURE 1:**
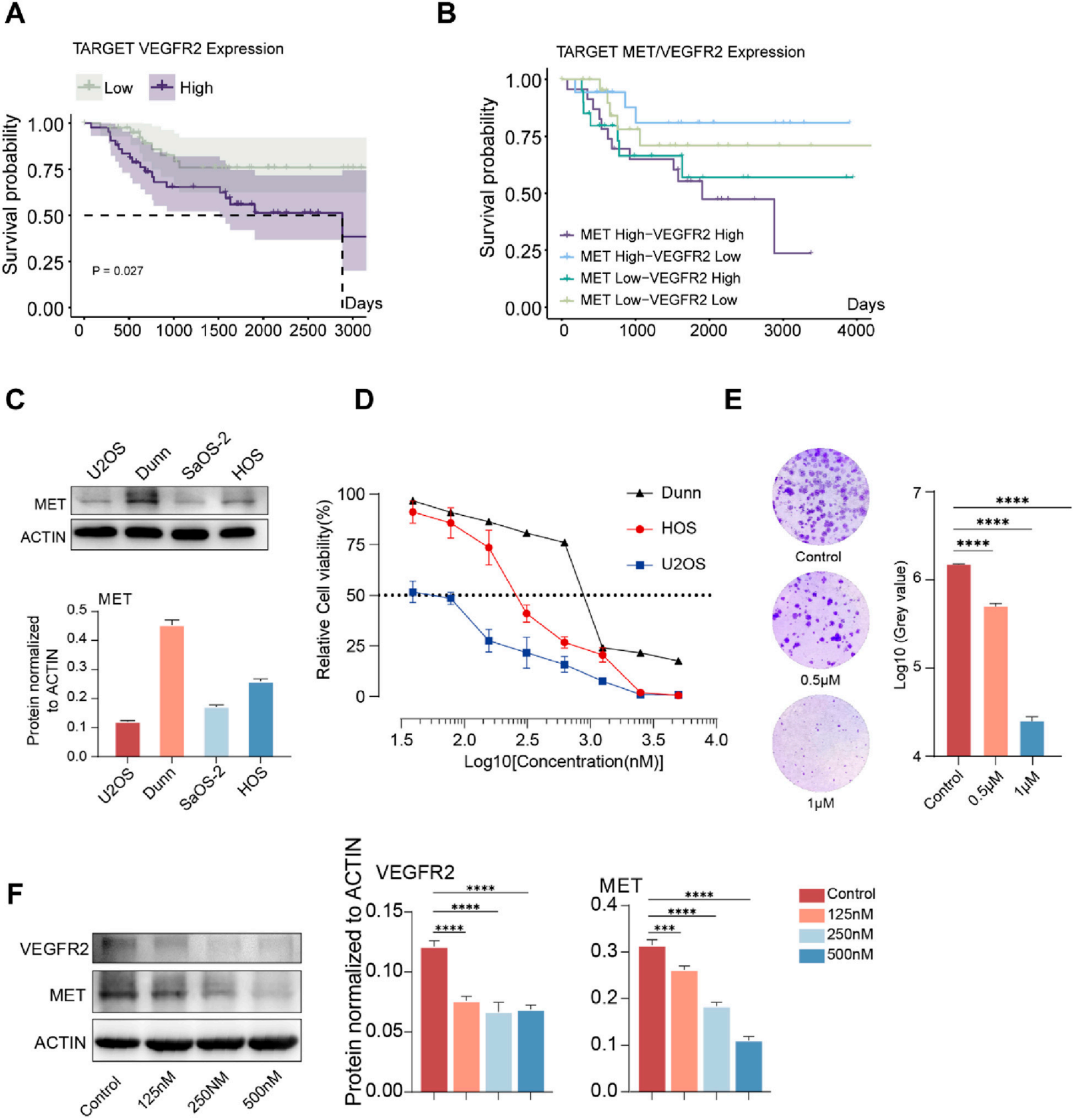
The degree of resistance to Anlotinib in osteosarcoma cells is positively correlated with the expression level of MET. **(A)**Kaplan-Meier curve of overall survival based on VEGFR2 expression level in the TARGET-OS cohort (n = 85). **(B)**Kaplan-Meier curve of overall survival based on MET and VEGFR2 expression level in the TARGET-OS cohort (n = 85). **(C)**Expression levels of MET in osteosarcoma cell lines U2OS, DUNN, SaOS-2, and HOS., **(D)** *In vitro* cell viability assay was conducted on osteosarcoma cell lines treated with Anlotinib, IC50: HOS 289.2 nM (95% CI: 242.2 nM–345.0 nM),U2OS 50.04 nM (95% CI: 41.55 nM–58.72 nM), 902.2 nM (95% CI: 770.4 nM−1058 nM) **(E)** *In vitro* clonogenic assay and quantitative analysis of the colony formation were performed among osteosarcoma cell lines treated with Anlotinib. **(F)** The protein expression levels and quantitative analysis of VEGFR2 and MET in HOS osteosarcoma cell after *in vitro* treatment with varying concentrations of Anlotinib. (***p < 0.001,****p < 0.0001).

We first analyzed whether the mRNA expression level of VEGFR2 was associated with the prognosis of patients in the TARGET-OS cohort and found that patients in the VEGFR2 high expression group had a shorter survival time (Figure 1A). We then analysed the impact of MET and VEGFR2 co-expression on patient outcomes, and the results of dual-gene survival analysis showed that patients in the MET and VEGFR2 co-high expression group had the worst prognosis (Figure 1B). These suggested that MET may co-cause disease progression with VEGFR2 in osteosarcoma.To verify whether MET is a factor contributing to Anlotinib resistance in osteosarcomacells, this study selected four osteosarcoma cell lines: U2OS, DuNN, SaOS-2, and HOS. Initially, the protein expression of MET was detected in these cell lines, revealing that MET was expressed in all 4 cell lines. Among them, DuNN exhibited the highest relative expression level of MET, while U2OS had the lowest (Figure 1C). Subsequently, gradient concentrations of Anlotinib were added to the systems of U2OS, DuNN, and HOS cell lines, and relative cell viability was measured after 72 h of culture to assess the efficacy of Anlotinib. The results indicated that the efficacy of Anlotinib was negatively correlated with the expression level of MET. Anlotinib had the weakest inhibitory effect on the cell line DuNN with the highest MET expression level, while it had the most significant effect on the cell line U2OS with low MET expression (Figure 1D). Furthermore, colony formation experiments were conducted by treating the HOS human osteosarcoma cell line with Anlotinib for 10 days. The results showed that the inhibitory effect of Anlotinib on cells increased as the concentration increased (Figure 1E). Additionally, we selected the HOS cell line, which had a median relative expression level of MET, and cultured it with gradient concentrations of Anlotinib. After 72 h, the protein expression levels of MET and VEGFR2 were detected (Figure 1F). The results showed that compared to the control group, the expression levels of MET and VEGFR2 were significantly downregulated in the Anlotinib-treated group. Taken together, these results suggest that the sensitivity of osteosarcoma cells to Anlotinib is related to their MET levels.

### 3.2 The combination of anlotinib and SCR1481B1 effectively enhances the inhibitory effect on tumor cells *in vitro*

**FIGURE 2:**
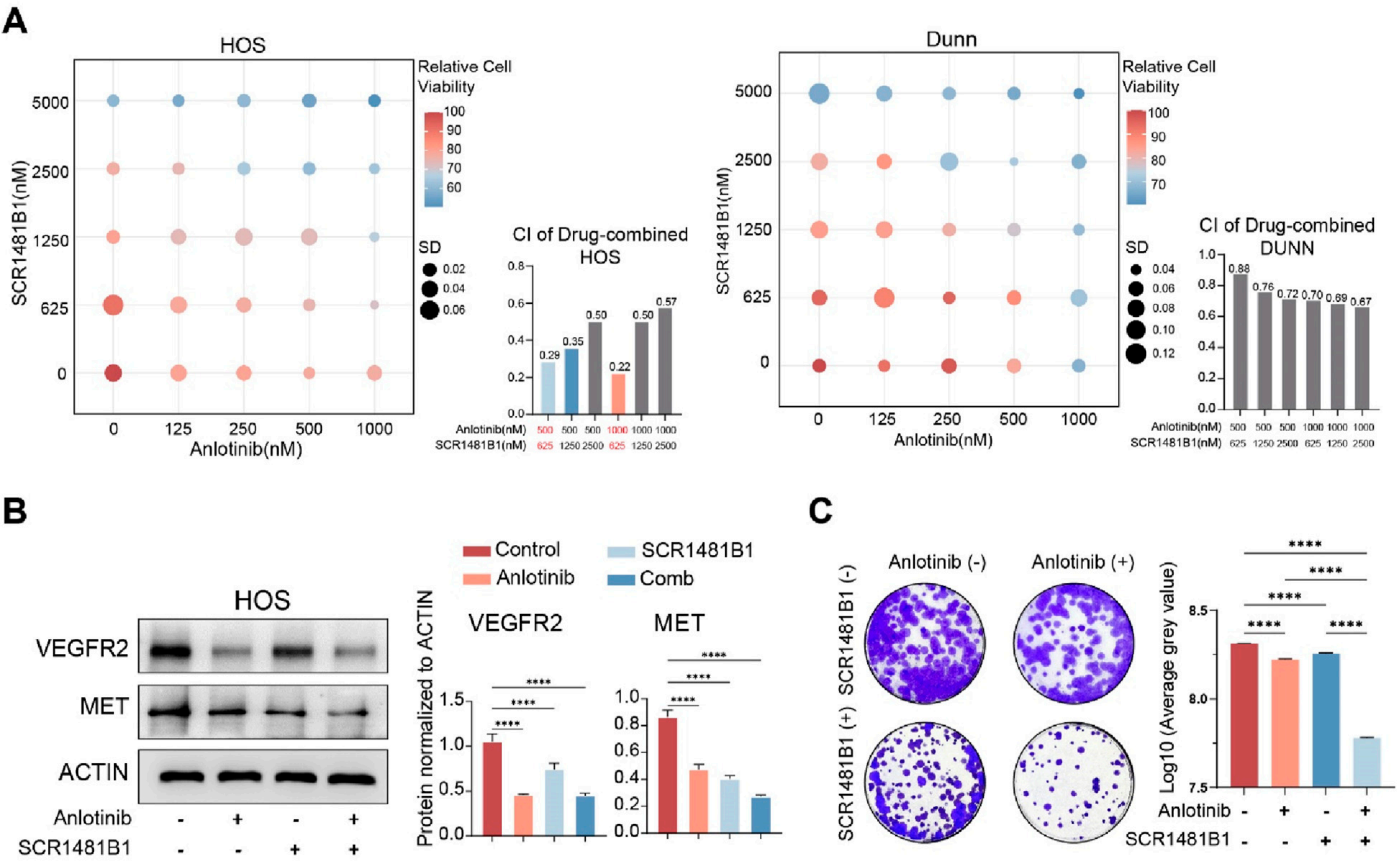
The combined use of Anlotinib and SCR1481B1 effectively enhances the inhibitory effect on tumor cells *in vitro*. **(A)** The combined application of Anlotinb and MET inhibitor (SCR1481B1) exhibits a synergistic effect on HOS and DuNN osteosarcoma cells. **(B)** The protein expression levels and quantitative analysis of VEGFR2 and MET in HOS osteosarcoma cell after *in vitro* treatment with the combination of Anlotinb and MET inhibitor (SCR1481B1) . **(C)** *In vitro* clonogenic assay and quantitative analysis of the colony formation were performed among osteosarcoma cell lines treated with the combination of Anlotinb and MET inhibitor (SCR1481B1). (****p < 0.0001).

Based on these findings, we hypothesized that there is a potential to enhance the VEGFR-targeted therapeutic effect by combining MET inhibitors. SCR1481B1, a MET inhibitor, is capable of acting on cancers that rely on MET activation. In this study, we selected 2 cell lines, HOS and DuNN, to verify our hypothesis. We treated osteosarcoma cells with combinations of SCR1481B1 and Anlotinib at different gradient concentrations for 72 h and then measured cell viability (Figure 2A). The results showed that as the concentration of the drug combination increased, the inhibitory effect on cell activity became more pronounced. When analyzing the correlation between the inhibitory effects of the two drugs on tumor cells, we found that the combination of Anlotinib and SCR1481B1 at concentrations of 500&625nM and 1000&625 nM had a strong synergistic killing effect on the tumor (CI < 0.3). Furthermore, Western blot analysis revealed that the expression levels of MET and VEGFR were significantly downregulated under the combined treatment of SCR1481B1 and Anlotinib (Figure 2B). Finally, colony formation experiments demonstrated that the combination of Anlotinib (concentration) and SCR1481B1 (concentration) could significantly inhibit the proliferation of osteosarcoma cells *in vitro* compared to the use of each drug alone after 10 days of treatment. As the concentrations of both drugs increased, the killing effect on tumor cells also correspondingly enhanced (Figure 2C).

### 3.3 The manufacturing process of magnetically driven hydrogel robots for drug delivery

**FIGURE 3:**
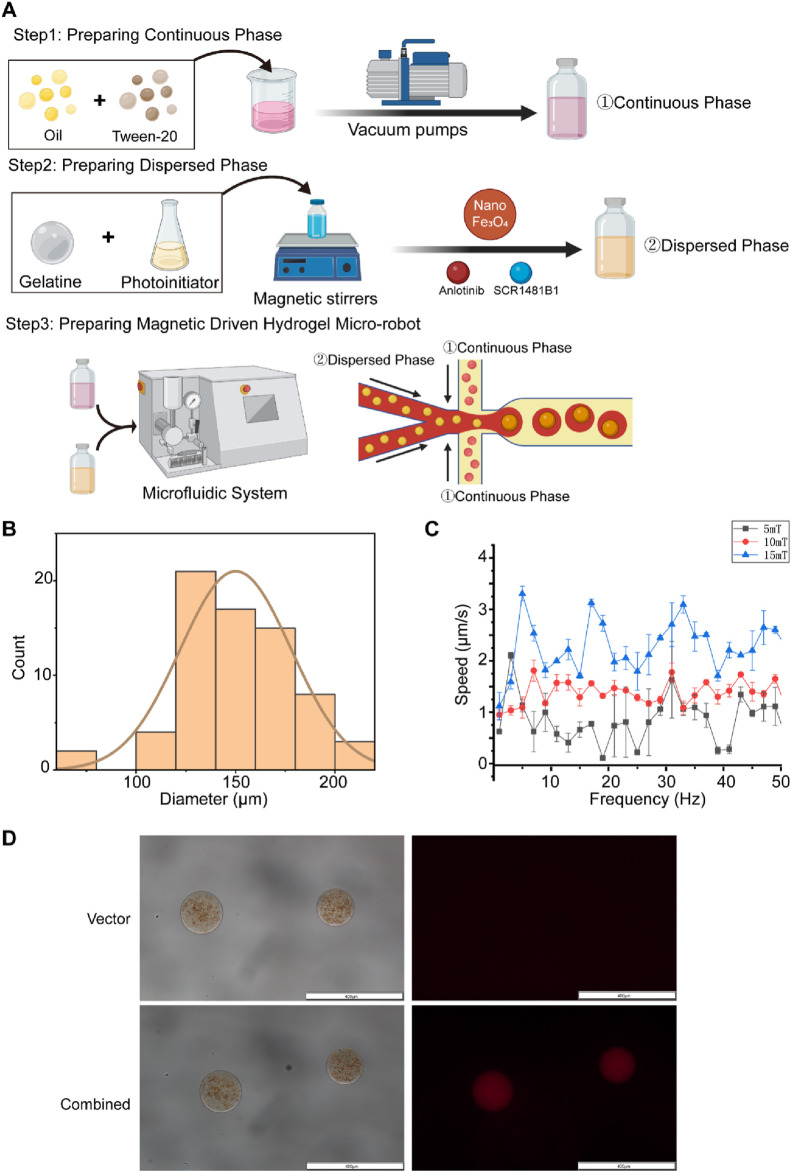
The orchestration of a targeted drug conveyance mechanism facilitated by the utilization of magnetic-driven hydrogel microrobots. **(A)** This study utilizes droplet microfluidic technology to construct gelatin microrobots. In the realm of droplet microfluidics, droplet formation is primarily governed by the interplay of tension differences between the continuous and dispersed phase fluids. The combined effect of surface tension and shear forces at the interface of these two phases gives rise to droplets. The accompanying figure presents a schematic representation of the fabrication of gelatin microspheres via a flow-focusing device. Created with BioRender.com. **(B)** Size distribution chart of Magnetic-driven hydrogel microrobot particles. **(C)** Motion rate characteristics of Magnetic-driven hydrogel microrobots at frequencies from two to 52Hz under different magnetic field densities. **(D)** Optical and fluorescence microscopy images of magnetic-driven hydrogel microrobots loaded with or without Anlotinib and MET inhibitor (SCR1481B1). The scale bar is 400 μm.

Based on the sensitization effect of SCR1481B1 on Anlotinib’s cytotoxicity against osteosarcoma cells, we have designed a magnetically driven microrobot loaded with both SCR1481B1 and Anlotinib. The objective is to carry the drugs, enabling them to precisely reach the target location under the influence of an external magnetic field and release the drugs through a delivery system, thereby enhancing the local therapeutic effect and reducing toxicological effects on normal tissues. In this study, we employed flow-focusing droplet microfluidics and emulsification methods to synthesize magnetically driven hydrogel microrobots loaded with SCR1481B1 and Anlotinib (Figure 3A). The magnetically driven hydrogel microrobots are manufactured using a microfluidic chip based on the flow-focusing principle. Briefly, we integrated the dispersive phase fluid containing SCR1481B1, Anlotinib, and Fe3O4 particles with the continuous phase that acts as a shaping and encapsulating agent. By experiencing shear forces between the liquids in a high-pressure narrow area, the two phases are combined to create magnetically loaded drug-bearing robots.We conducted a statistical analysis of the size distribution of the manufactured microrobots and found that the particle size distribution follows a normal distribution, with most microrobots having a diameter of approximately 150 μm (Figure 3B). After assembly of the magnetically driven hydrogel microrobots, we examined their movement under different magnetic field strengths. By adjusting the intensity and frequency of the magnetic field, the microrobots can achieve a peak speed of 3.5 μm/s (Figure 3C).In summary, our research results indicate that the magnetically driven hydrogel microrobots constructed in this study demonstrate controllable motion patterns. This ensured the structural integrity and magnetic responsiveness of the magnetically driven hydrogel microrobots while guaranteeing excellent drug release performance.

To evaluate the drug-loading characteristics and effects of the microrobots, we employed the fluorescent properties of Rhodamine B by co-adding it with the drugs into the central dispersive phase. We then used a fluorescence microscope to examine the characteristics of the combined drug loading (Figure 3D). Under bright-field visualization, unloaded magnetically driven hydrogel microrobots appeared brown and did not display fluorescence. Simultaneously, magnetically driven hydrogel microrobots loaded with SCR1481B1 and Anlotinib did not show significant differences from other components under bright-field conditions, but red fluorescence could be observed within the structure of the microrobots under a fluorescence microscope. The experimental results indicate that both SCR1481B1 and Anlotinib have been successfully integrated into the microrobots.

### 3.4 Magnetically driven microrobots possess the characteristics of precise and controlled guided motion as well as sustained drug release

**FIGURE 4:**
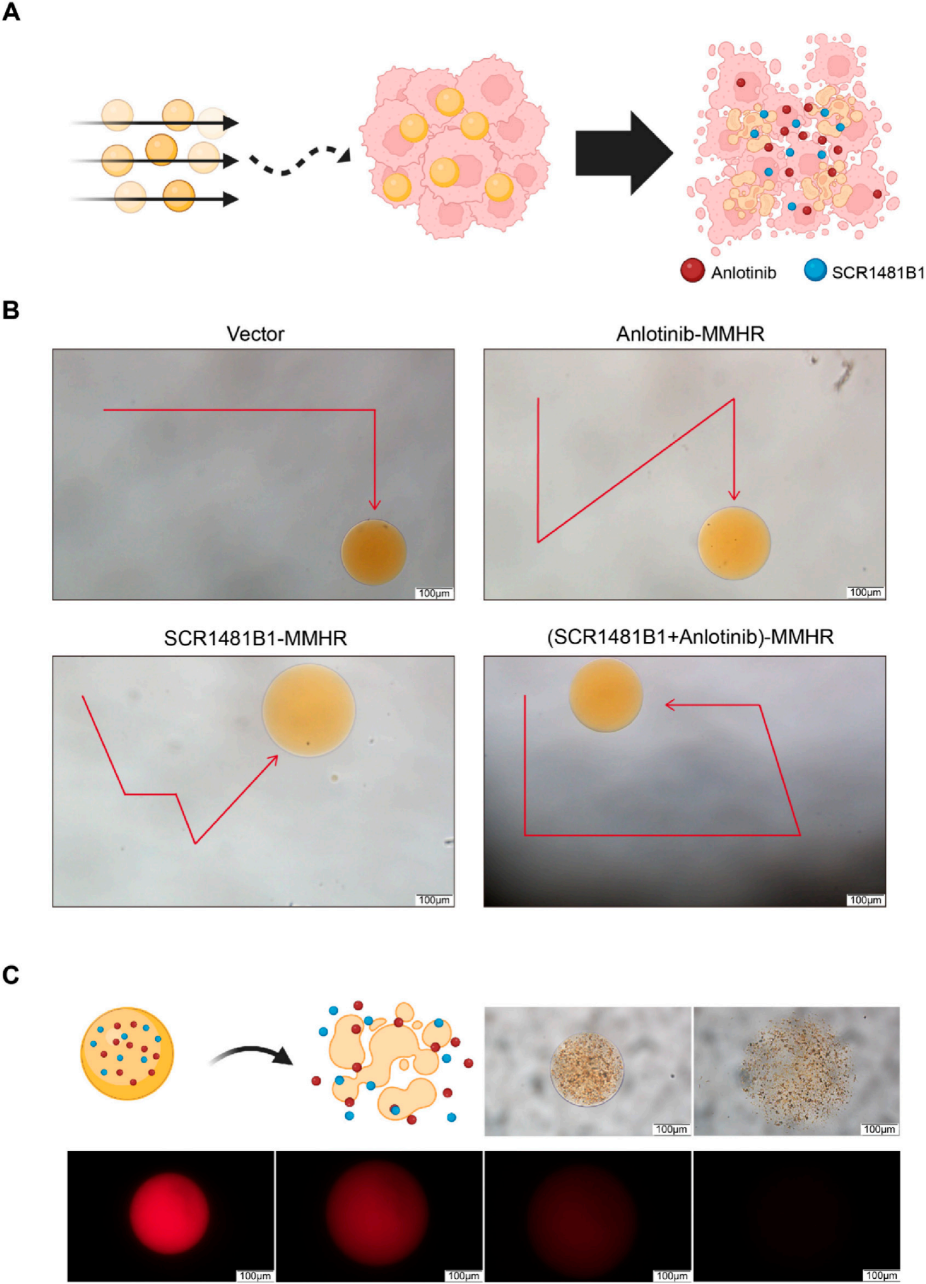
Motion characteristics, controllable flexible motion performance and drug release performance of drug-loaded Magnetic-driven hydrogel microrobots. **(A)**Schematic illustration of tumor destruction mediated by a drug-loaded delivery system facilitated by magnetic-driven hydrogel microrobots. Created with BioRender.com **(B)** Controllable motion modes of Magnetic-driven hydrogel microrobots without drug loading. Controllable motion modes of Anlotinb-loaded Magnetic-driven hydrogel microrobots. Controllable motion modes of SCR1481B1-loaded Magnetic-driven hydrogel microrobots. Controllable motion modes of Magnetic-driven hydrogel microrobots loaded with Anlotinib and SCR1481B1. The scale bar is 100 μm. **(C)** Schematic illustration of drug release from drug-loaded Magnetic-driven hydrogel microrobots (Created with BioRender.com) and optical microscopy and fluorescence microscopy images of drug release from drug-loaded Magnetic-driven hydrogel microrobots. The scale bar is 100 μm.

Whether it is possible for micro- and nano-robots to accurately deliver drugs to tumor sites and effectively release them is the key to DDS design. The controllable motion of the drug delivery system and its effective drug release capability are important indicators for evaluating the effectiveness of the drug delivery system. We hope to design robots with precise navigation capabilities that can reach tumor sites and efficiently release loaded drugs, thereby enhancing the tumor killing effect (Figure 4A). Therefore, we first examined the motion patterns of our designed magnetically driven hydrogel microrobots loaded with drugs by applying an externally controllable magnetic field. By adding magnetic fields with different vectors on a plane, fine-tuned directional motion of the magnetically driven hydrogel microrobots can be achieved (Figure 4B). By changing the direction of the magnetic field, controlled and regular motion trajectories of magnetically driven hydrogel microrobots loaded with or without drugs. (Supplementary Videos S1-4).

Subsequently, to verify the drug release effect of magnetically driven hydrogel robots, we monitored the drug release at different time intervals under both bright-field and fluorescence microscopes. We observed that the robots gradually degraded over time while releasing the loaded drugs. After 15 min, all drug-loaded magnetically driven hydrogel microrobots exhibited consistent drug release capabilities (Figure 4C). The above experimental results indicate that under the control of an external magnetic field, hydrogel microrobots can perform precise motion to enhance the targeted drug delivery capability and efficiently release drugs after reaching the navigation site, providing critical support for precise drug delivery.

### 3.5 The magnetically driven hydrogel microrobots loaded with SCR1481B1 and anlotinib exert a killing effect on osteosarcoma cells *in vitro*

**FIGURE 5:**
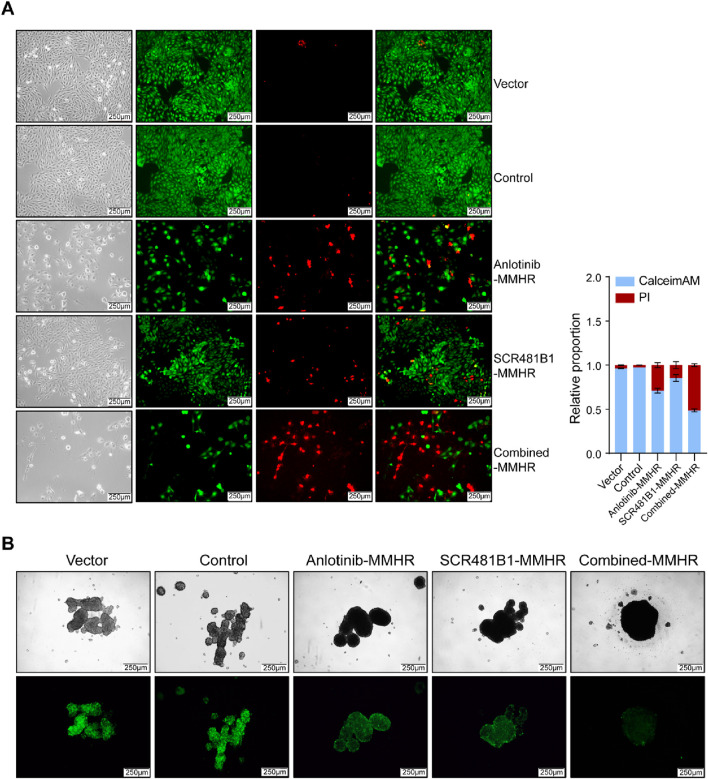
Drug-loaded Magnetic-driven hydrogel microrobots exhibit potent cytotoxicity against osteosarcoma cells (under both two-dimensional and three-dimensional cell culture conditions) through the release of drugs. **(A)** Representative optical and fluorescence microscope images of osteosarcoma cells under two-dimensional culture conditions following a combined treatment regimen with Anlotinb and SCR1481B1. Calcein/AM-PI double staining was conducted to detect the cytotoxic effects exerted by both unloaded Magnetic-driven hydrogel microrobots and drug-loaded Magnetic-driven hydrogel microrobots on osteosarcoma cells. The magnification is 100 times. The scale bar is 250 μm. **(B)** Representative optical and fluorescence microscope images of osteosarcoma cells under three-dimensional culture conditions after combined treatment with Anlotinb and SCR1481B1. The magnification is 100 times. The scale bar is 250 μm.

Finally, we simulated the antitumor effect of the magnetically driven hydrogel micro-robot drug delivery system *in vitro*. We placed them in the center of culture dishes under both two-dimensional (2D) and three-dimensional (3D) cell culture conditions. For cells cultured in 2D conditions, we used calcein-AM/PI double staining to distinguish dead cells 72 h after drug release (Figure 5A). For cells cultured in 3D conditions, we measured the expression of green fluorescence to assess the toxic effects of the drugs on tumor cells (Figure 5B). The experimental results demonstrated that the magnetically driven hydrogel microrobots loaded with both SCR1481B1 and Anlotinib exhibited excellent tumor killing effects on tumor cells under both 2D and 3D culture conditions. Compared with the control group, the addition of unloaded robots did not significantly affect tumor cell growth, indicating that the material itself of our designed robots had no killing effect on tumor cells. In contrast, the robots loaded with both SCR1481B1 and Anlotinib exhibited superior tumor killing effects compared to those loaded with a single drug. Our *in vitro* experiments showed that the magnetically driven microrobots loaded with SCR1481B1 and Anlotinib can effectively deliver drugs while enhancing the killing effect on tumor cells.

## 4 Discussion

The aim of this study is to explore the therapeutic potential of combining MET inhibitors with Anlotinib for the treatment of Anlotinib-resistant osteosarcoma. We designed and produced magnetically driven hydrogel microrobots loaded with drugs, and by incorporating SCR1481B1, we aimed to enhance the sensitivity of osteosarcoma cells to Anlotinib. Through two-dimensional and three-dimensional cell culture models, we have demonstrated that our magnetically driven hydrogel micro-robot-based drug delivery system can can guide controlled movement to the target site and sustain drug release *in vitro* therefore enhancing the sensitivity of Anlotinib-resistant osteosarcoma cells.

Drug delivery systems (DDS) refer to technological systems that comprehensively regulate the distribution of drugs within the body in terms of space, time, and dosage. They address issues related to controlled drug release and targeted delivery. By adjusting the delivery and release location of drugs, these systems can alter the metabolic behavior of drugs within the body, overcome physiological barriers to enhance drug absorption, increase the bioavailability of drugs, improve therapeutic efficacy, and simultaneously reduce toxic and side effects (Yang et al., 2022). Magnetically driven hydrogel microrobots (MMHR) consist of hydrogel filled with nanometer-sized magnetic particles. When an external magnetic field is applied, the magnetic particles dispersed within the hydrogel receive magnetic forces and transmit them to the hydrogel matrix, guiding the directional movement of the robot. This allows for the loading of large amounts of drugs, making them suitable for drug delivery (Florea, 2020). MMHR as DDS possess unique advantages. Firstly, compared to traditional rigid robots, using hydrogel as the main scaffold material of the robot endows the soft robot with a highly deformable body and high degrees of freedom. The smaller difference in elastic modulus between the robot components and living tissue reduces safety hazards, making it more compliant and safe within the human body. Secondly, MMHR can utilize the high water absorption and porosity of hydrogel to achieve effective drug loading and responsive drug release, thus avoiding premature drug leakage and nonspecific effects. Finally, MMHR can leverage the biocompatibility and biodegradability of hydrogel to minimize toxicity and side effects on the organism.

Osteosarcoma is a type of malignant tumor that occurs in the bones and primarily affects children and adolescents. The main treatment strategies for osteosarcoma include surgical resection and chemotherapy. However, due to the significant heterogeneity and drug resistance of osteosarcoma, the efficacy of chemotherapy is often not ideal, leading to high recurrence and metastasis rates. At the same time, the complex genetic background of osteosarcoma also poses difficulties for targeted therapy. Targeting VEGFR is a long-standing tumor treatment strategy that aims to inhibit tumor angiogenesis and malignant cell proliferation by blocking VEGFR. Over the years, VEGFR-targeted drugs have been widely and successfully used in the treatment of various solid tumors, and new drugs represented by Anlotinib have been continuously developed on this basis. Nevertheless, the high heterogeneity and complex genetic characteristics of osteosarcoma prevent some patients from benefiting from relevant targeted drugs. High expression of the MET gene is considered an independent factor for poor prognosis in tumors. MET, as a proto-oncogene, can act as an independent carcinogenic factor to drive cancer development. When MET is amplified or overexpressed, cells continuously receive proliferative signals, leading to sustained tumor growth. Additionally, MET can mutate during tumorigenesis, acting as a secondary factor that contributes to adverse events such as drug resistance (Bradley et al., 2017). Our experimental results demonstrate that the combined use of MET inhibitors in osteosarcoma may be a strategy to enhance the killing effect of Anlotinib. Unfortunately, there is currently no available Anlotinib-resistant cell line to further establish a drug resistance model *in vitro* to validate our experimental results.

By designing magnetic hydrogel microrobots loaded with both Anlotinib and SCR1481B1, we have established an effective drug delivery system. We have confirmed the sensitivity of MET-overexpressing osteosarcoma cells to MET inhibitors *in vitro* and successfully enhanced their response to Anlotinib. Therefore, our magnetic hydrogel microrobots drug delivery system shows promise for treating Anlotinib-resistant osteosarcoma. However, in a clinical setting, osteosarcoma patients often have a substantial tumor burden, and the flow rate of blood and the viscosity of plasma pose challenges for the microrobots to navigate in liquid media. Whether the microrobots can overcome blood flow resistance to reach the tumor site and penetrate the primary tumor to deeper region are questions worthy of further investigation. At present, most of the research on medical actively driven microrobots is limited to *in vitro* simulation experiments due to the complex *in vivo* environmental conditions of humans or experimental animals and the limitations of robot performance (Yu et al., 2023; Zhang et al., 2024). Encouragingly, T. Li and his team have further improved the driving efficiency of micro-nano robots by designing a claw-shaped surface structure that mimics the movement of tardigrades (water bears) using their claws in dynamic environments, enabling them to resist blood flow impacts and navigate through blood vessels (Li et al., 2023). Inspired by this, we are actively trying to improve the driving characteristics of the robot while retaining its ability to carry drugs as DDS, so that it can travel stably in high-velocity venous blood flow to the tumor site. And select a suitable animal model to verify the pharmacokinetics of the drug-loaded robot, and use appropriate imaging methods to image the nanorobot particle swarm to verify the robot’s active targeting effect.In order to validate whether magnetically-driven hydrogel microbots capable of drug delivery can effectively enhance the killing effect on osteosarcoma under the guidance of genomic characteristics.

Efficient drug delivery systems (DDS) are used in cancer treatment with the aim of precisely delivering drugs to tumor sites, thereby minimizing toxicological effects on normal tissues. DDS holds significant research value in tumor therapy, as they can provide new strategies and methods for treating various types of tumors, thereby improving the survival rate and quality of life for cancer patients. Micro drug-loaded robots as a promising drug delivery system (DDS) the application of DDS in cancer treatment still faces some challenges. For instance, improving robot’s driving capabilities, stability, biocompatibility, drug loading, and drug release rate are urgent issues that need to be addressed in the design and optimization of DDS. Additionally, effectively and accurately identifying relevant oncogenes and their specific mechanisms that play a synergistic role in tumors can lead to the development of more effective combination targeted therapy strategies for treating various drug-resistant osteosarcomas, thereby improving the quality and survival rate of patients. With advancements in the diagnosis and treatment of osteosarcoma, as well as the deepened research on its pathogenesis through genomics and other methods, future osteosarcoma treatment strategies will undoubtedly become more refined and individualized, given the complex genetic background and heterogeneity of the disease. The refined selection of chemotherapy, surgery, gene therapy, and immunotherapy will undoubtedly cover a wider range of osteosarcoma patients more effectively. DDS and combined targeting have demonstrated significant research value in the treatment of various tumors, offering new insights and methods for precision and individualized therapy for osteosarcoma patients.

## Data Availability

The raw data supporting the conclusions of this article will be made available by the authors, without undue reservation.
