# Supplementary figures and images for: Magnetically driven hydrogel microrobots for enhancing the therapeutic effect of anlotinib on osteosarcoma
Source: Front Bioeng Biotechnol. 2024 Nov 13;12:1409988. doi: 10.3389/fbioe.2024.1409988 (PMC11600141; doi:10.3389/fbioe.2024.1409988)

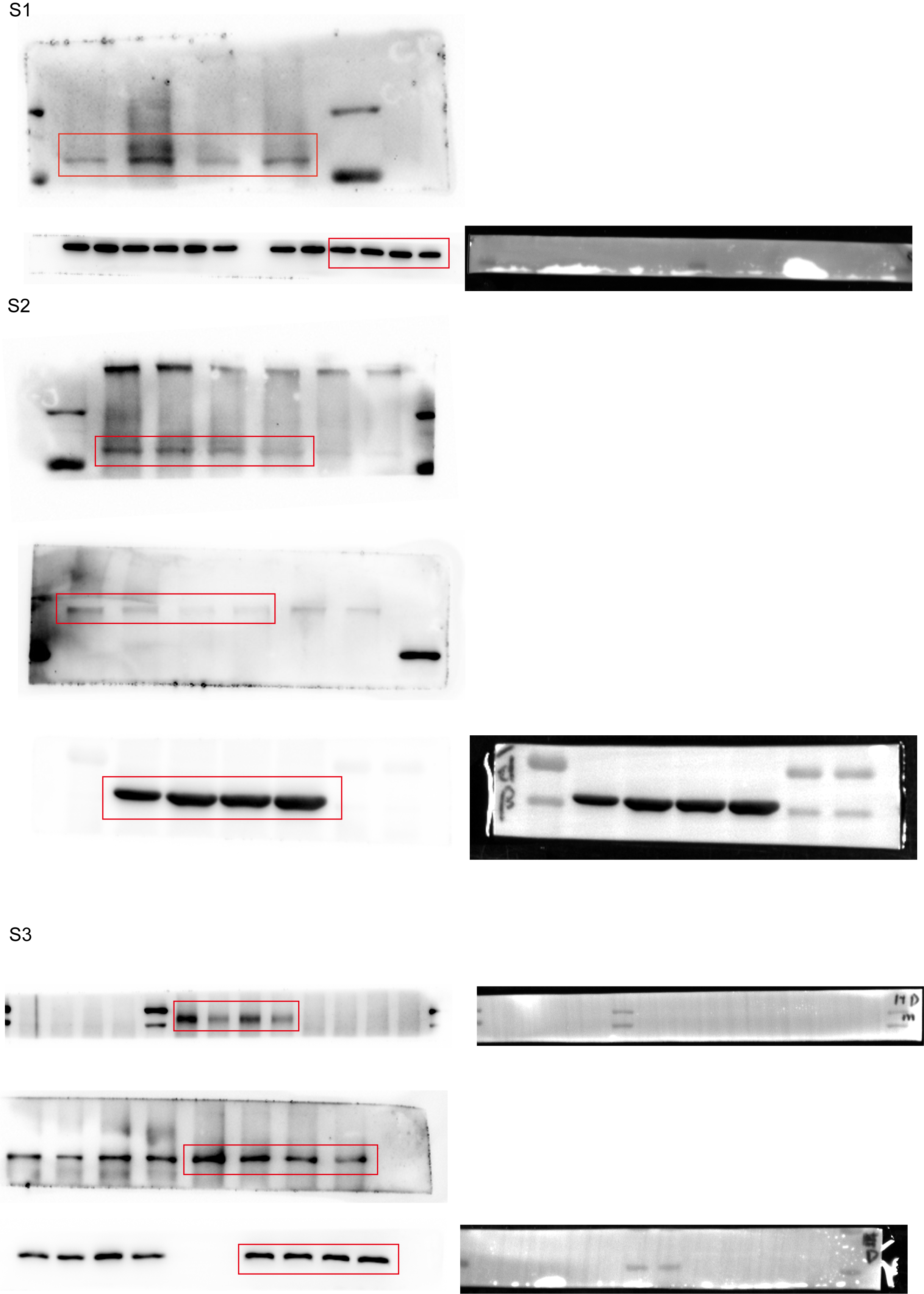

Supplement: Supplementary file 4 [file Image1.PNG]
